# Decreased Cdk2 Activity Hindered Embryonic Development and Parthenogenesis Induction in Silkworm, *Bombyx mori* L.

**DOI:** 10.3390/ijms26073341

**Published:** 2025-04-03

**Authors:** Chengjie Hu, Yonghou Jiang, Chenkai Ma, Fang Xu, Chunguang Cui, Xin Du, Jine Chen, Linbao Zhu, Shaofang Yu, Xingjian He, Wei Yu, Yongqiang Wang, Xia Xu

**Affiliations:** 1 College of Life Sciences and Medicine, Zhejiang Sci-Tech University, Hangzhou 310018, China; 2 Institute of Sericulture and Tea, Zhejiang Academy of Agricultural Sciences, Hangzhou 310021, China

**Keywords:** Cdk2, AUZ454, oocytes, embryo, silkworm

## Abstract

Cyclin-dependent protein kinase 2 (Cdk2), an important member of the serine/threonine-specific protein kinase family, plays a critical regulatory role in biological processes. Previous studies have demonstrated that Cdk2 is involved in the arrest and resumption of meiosis in mammalian oocytes. In this study, we explored the function of Cdk2 through parthenogenetic lines (PLs) and corresponding amphigonic lines (ALs) in a model lepidopteran insect silkworm, *Bombyx mori* L. Our findings revealed a positive correlation between Cdk2 activity and the parthenogenesis induction rate. The pharmacological inhibition of Cdk2 using the specific inhibitor AUZ454 not only significantly reduced the parthenogenesis induction rate but also caused developmental delays in embryos. These results demonstrate that Cdk2 is essential for parthenogenesis success and is a potential target gene for biological reproductive regulation.

## 1. Introduction

Parthenogenesis is when an egg develops into a new normal individual without fertilization, which is widespread in nature, especially in insects [1,2,3]. This mechanism serves as an adaptive response, enabling species to survive and proliferate in challenging environments [4,5,6]. Beyond naturally occurring parthenogenesis, artificial parthenogenesis induction has been successfully achieved in diverse organisms, including algae, fish, pigs, and silkworms [7,8,9,10]. In the model lepidopteran insect *Bombyx mori* L., which typically reproduces sexually, spontaneous parthenogenesis occurs at an extremely low frequency (~0.003%) [11,12]. However, the thermal stimulation (46 °C, 18 min) of unfertilized silkworm eggs can induce parthenogenesis, yielding genetically identical female progeny [13,14]. Therefore, it has a high value in the field of genetics and breeding.

Based on the cytological mechanism of embryonic development, parthenogenesis is classified into two types: ameiotic parthenogenesis (AMP) and meiotic parthenogenesis (MP) [15]. AMP is an oocyte that does not undergo complete meiosis and retains diploid, undergoing a process similar to mitosis [16]. MP enables oocytes to undergo meiosis and restore diploid through gametic replication, terminal fusion, central fusion, and other mechanisms [17]. In silkworm, oocytes initiate meiosis I upon ovulation but arrest at metaphase I, requiring sperm-derived or artificial stimuli to resume division [18]. The warm bath induced the silkworm to belong to AMP, which destroyed the formation of a spindle and resulted in the early separation of homologous chromosomes. Subsequently, a reconstituted spindle with an equatorial plate develops within the oocyte, its components no longer bind, and the oocyte only divides equally once to produce a diploid prokaryote [19]. These events involve intricate cell cycle modifications, highlighting the critical role played by cell cycle regulation in parthenogenetic development [20].

Cyclin-dependent protein kinase 2 (Cdk2), a serine/threonine-specific protein kinase, binds to specific cyclin partners to regulate essential processes such as cell cycle, division, and metabolism [21,22,23]. During the development and meiosis of animal oocytes, Cdk2 precisely regulates the cell cycle process to ensure that oocytes stop and resume division at the right time [24]. In *Mus musculus* L., Cdk2 knockout disrupts the pachytene-to-diplotene transition in prophase I, leading to oocyte death [25]. In *Sus scrofa domesticus* L., blocking Cdk2 activity caused the oocytes to fail to enter the second stage of meiosis [26]. In *Xenopus laevis* D., the inhibition of Cdk2 activation led to the secondary meiosis arrest failure of mature oocytes [27]. Cdk2 functions by phosphorylating specific protein substrates, and loss of its catalytic activity disrupts gametogenesis [28]. Moreover, Cdk2 activity was also essential for correct spindle and chromosome dynamics. Its inhibition of activity led to the continuous activation of the spindle assembly checkpoint (SAC) and thus prevented the meiosis process [29].

In this study, we found that the mRNA expression of Cdk2 was significantly higher in parthenogenetic lines (PLs) and corresponding amphigonic lines (ALs) after warm bath treatment in silkworm. Subsequently, we investigated the role of Cdk2 in silkworm parthenogenesis using the selective inhibitor AUZ454. The inhibition of Cdk2 activity was not toxic to silkworm eggs but hindered embryo development, resulting in a decrease in the parthenogenesis induction rate. These results indicated the importance of Cdk2 for the artificial induction of silkworm parthenogenesis. In addition, Cdk2 was evolutionarily highly conserved and could be a potential target for insect reproductive regulation.

## 2. Results

### 2.1. Protein Structure and Phylogenetic Identification of Cdk2

**Figure 1:**
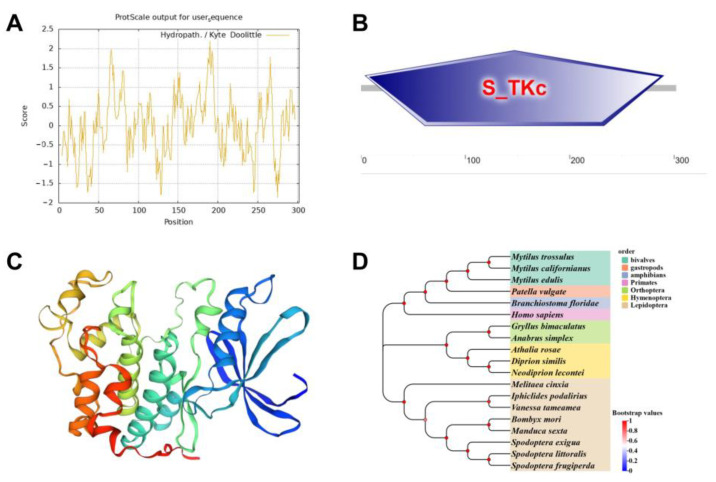
Predicted protein and phylogenetic analysis. (**A**) Physicochemical properties. (**B**) Functional domain. The S_TKc region was the catalytic domain of serine/threonine protein kinases. (**C**) Tertiary structure. (**D**) Phylogenetic tree. Protein sequences from *Bombyx mori* L. (NP 001266420.1), *Melitaea cinxia* L. (XP 045449529.1), *Iphiclides podalirius* L. (CAH2040048.1), *Vanessa tameamea* E. (XP 026500363.1), *Manduca sexta* B. (XP 030023086.1), *Spodoptera exigua* F. (CAD0244601.1), *Spodoptera littoralis* B. (CAB3505944.1), *Spodoptera frugiperda* S. (XP 035441455.1), *Athalia rosae* L. (XP 012260508.1), *Diprion similis* H. (XP 046741319.1), *Neodiprion lecontei* F. (XP 015518064.1), *Gryllus bimaculatus* G. (GLH03008.1), *Anabrus simplex* H. (XP 066991469.1), *Mytilus trossulus* G. (XP 063423120.1), *Mytilus californianus* C. (XP 052094668.1), *Mytilus edulis* L. (CAG2221041.1), *Patella vulgate* L. (XP 050404957.1), *Branchiostoma floridae* H. (XP 035658594.1), and *Homo sapiens* L. (CAA43807.1).

The Cdk2 protein comprises 302 amino acids with a theoretical isoelectric point (pI) of 8.77, exhibiting strong hydrophilicity due to its predominantly polar residues (Figure 1A). The catalytic serine/threonine protein kinase domain (aa 4–290) was highly conserved (Figure 1B,C). Homologous sequences of the Cdk2 protein were selected from different representative species to explore evolutionary conservation. The sequences evaluated were from lepidoptera (*B. mori* L., *Melitaea cinxia* L., *Iphiclides podalirius* L., *Vanessa tameamea* E., *Manduca sexta* B., *Spodoptera exigua* F., *Spodoptera littoralis* B., *Spodoptera frugiperda* S.), hymenoptera (*Athalia rosae* L., *Diprion similis* H., *Neodiprion lecontei* F.), Orthoptera (*Gryllus bimaculatus* G., *Anabrus simplex* H.), Bivalvia (*Mytilus trossulus* G., *Mytilus californianus* C., *Mytilus edulis* L.), Gastropoda (*Patella vulgate* L.), Amphibian (*Branchiostoma floridae* H.), and Primates (*Homo sapiens* L.). The Cdk2 protein sequence was evolutionarily highly conserved (Figure 1D). These data indicate that findings in *B. mori* regarding the function of Cdk2 are likely applicable to other species.

### 2.2. Differences in Cdk2 mRNA Expression and Activity Between PLs and ALs

**Figure 2:**
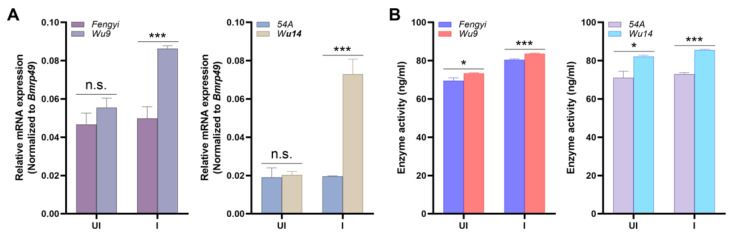
The mRNA expression and activity of Cdk2. (**A**) mRNA expression. (**B**) Enzyme activity. Parthenogenetic lines: Wu9, Wu14; corresponding amphigonic lines: Fengyi, 54A. The mRNA expression level was normalized to *B. mori ribosomal protein 49* (*Bmrp49*), an internal reference. The data shown are means ± S.E.M. (*n* = 3). Asterisks indicate significant differences with a two-tailed *t*-test: * *p* < 0.05; *** *p* < 0.001; n.s. *p* > 0.05.

Unfertilized eggs of female moths were taken 12 h after emergence, some of which were directly collected as uninduced eggs (UI), and some were immediately collected as induced eggs (I) after warm bath. We detected *Cdk2* mRNA expression levels in both UI and I. The results showed that there was no significant difference between PLs (Wu9, Wu14) and corresponding ALs (Fengyi, 54A) in UI, but there were extremely significant differences in I (Figure 2A). We subsequently detected the Cdk2 activity. Similarly, the Cdk2 activity was significantly different in UI and extremely significantly different in I (Figure 2B). These results suggested that Cdk2 played an important role in the silkworm parthenogenesis induction.

### 2.3. AUZ454 Effectively Inhibited Cdk2 Activity and Was Non-Toxic

**Figure 3:**
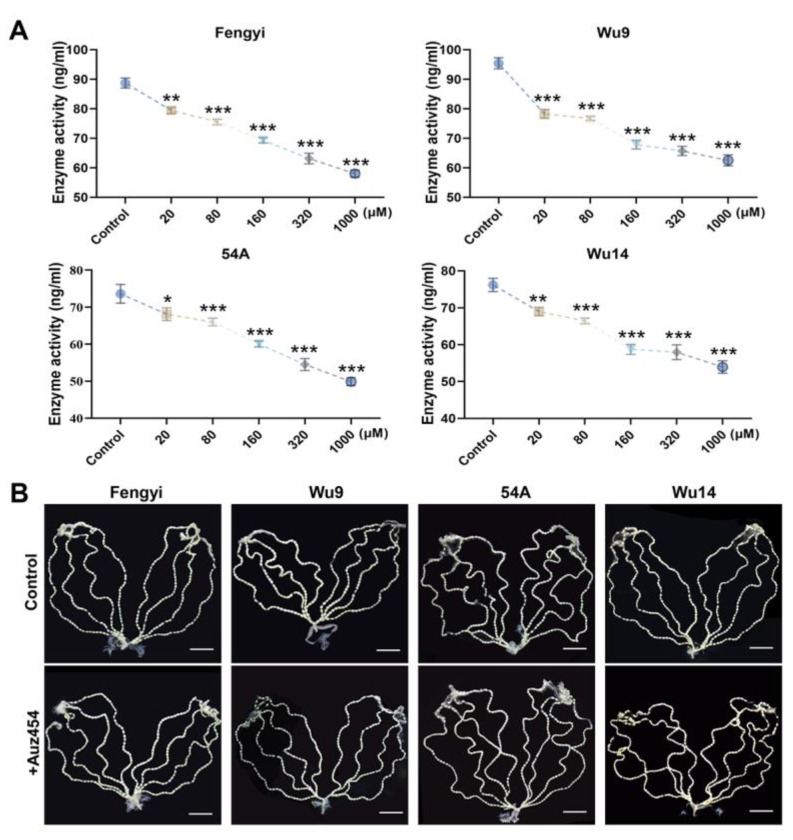
AUZ454-inhibited Cdk2 activity. (**A**) Enzyme activity. (**B**) Ovarioles observation. Parthenogenetic lines: Wu9, Wu14; corresponding amphigonic lines: Fengyi, 54A. The scale was 1 cm. The data shown are means ± S.E.M. (*n* = 3). Asterisks indicate significant differences with a two-tailed *t*-test: * *p* < 0.05; ** *p* < 0.01; *** *p* < 0.001.

Twelve hours after pupae emergence, female moths were injected with the Cdk2 inhibitor AUZ454 at varying concentrations. Following treatment, unfertilized eggs were collected and analyzed for Cdk2 activity. We found that the enzyme activity was significantly inhibited and decreased significantly as the concentration of the inhibitor increased (Figure 3A). Subsequently, we observed ovariole integrity and egg development as represented by the maximum concentration of interference. After inhibitor interference, the eight ovarioles were completely arranged on both sides of the abdomen, and there was no significant difference in the development status and shape of the eggs compared with the control (Figure 3B). These results indicated that AUZ454 specifically inhibits Cdk2 activity without causing adverse effects on ovarian structure or egg development.

### 2.4. Cdk2 Activity Affected Parthenogenesis Induction

**Figure 4:**
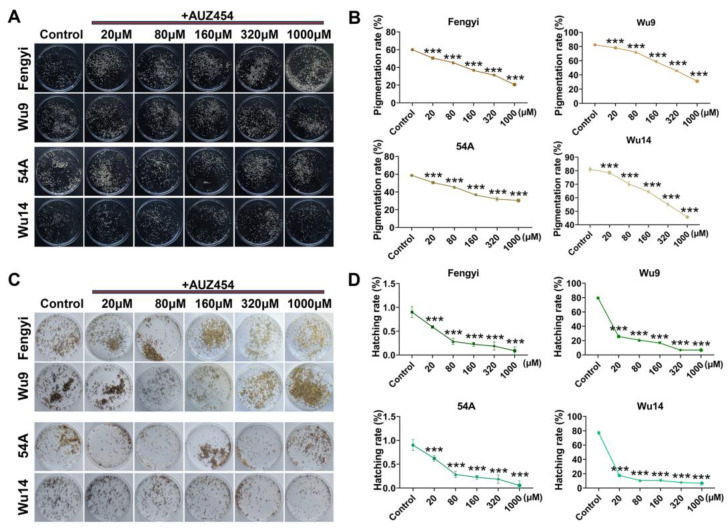
Cdk2 activity affected parthenogenesis. (**A**) Pigmentation eggs. (**B**) Pigmentation rates. (**C**) Hatching eggs. (**D**) Hatching rates. Parthenogenetic lines: Wu9, Wu14; corresponding amphigonic lines: Fengyi, 54A. Yellow: non-pigmentation egg, brown: pigmentation egg, white: hatching egg shell. The diameter of the circular petri dish was 9 cm. The data shown are means ± S.E.M. (*n* = 3). Asterisks indicate significant differences with a two-tailed *t*-test: *** *p* < 0.001.

Following inhibitor treatment, unfertilized eggs were subjected to warm bath induction (46 °C, 18 min) to initiate parthenogenesis and terminate diapause. The parthenogenesis induction was observed and calculated. When Cdk2 activity was not disturbed, the pigmentation rates of ALs were less than 60%, and those of PLs were more than 80%. The decrease in Cdk2 activity resulted in an extremely significant decrease in the pigmentation rates of Als and PLs (Figure 4A,B). Parallel effects were observed in hatching success, with control ALs and PLs showing approximately 1% and 80% hatching rates, respectively. Interference with enzyme activity resulted in a significant decrease in the hatching rate (Figure 4C,D). It was further indicated that Cdk2 activity was crucial for parthenogenesis induction.

### 2.5. Inhibition of Cdk2 Activity Hindered Embryonic Development

**Figure 5:**
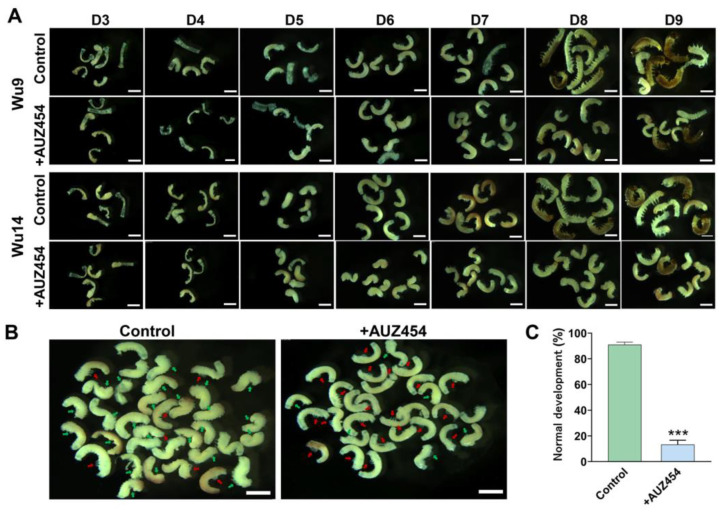
Interference with Cdk2 activity led to abnormal embryonic development. (**A**) The process of embryonic development. (**B**) Embryo of reversal stage. (**C**) Normal reversed embryo ratio. Green arrow: reversed embryo; red arrow: unreversed embryo. The scale was 1mm. The data shown are means ± S.E.M. (*n* = 3). Asterisks indicate significant differences with a two-tailed *t*-test: *** *p* < 0.001.

Embryonic development progressed through characteristic stages of tissue formation and organogenesis, culminating in complete larval development by day 9. We observed the embryonic development of PLs (Wu9 and Wu14) from the visible formation stage of blastodermal differentiation on day 3 to the complete stage of full development on day 9. We found that after the inhibitor interference, embryonic development was delayed, especially during the embryo reversal stage on day 6 (Figure 5A). In the normal reversal stage, embryos undergo a 180° rotation, transitioning from their initial ventral orientation to assume a dorsal position facing the egg center. In the controls, more than 90% of embryos had completed reversal, while only 10% had normal reversal after inhibitor interference (Figure 5B,C). Cdk2 activity affected the success rate of parthenogenesis induction and delayed or even stopped embryonic development.

### 2.6. Knockdown of Cdk2 Gene Affected G1/S Cell Cycle Transition

**Figure 6:**
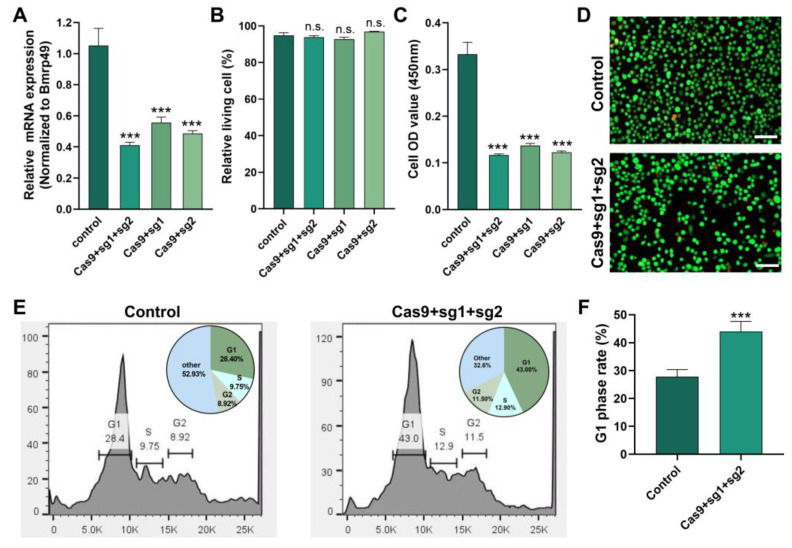
Knockdown of *Cdk2* gene affected cell cycle. (**A**) The mRNA expression of *Cdk2* gene. (**B**) Cell viability. (**C**) Cell proliferation. (**D**) Cell fluorescence staining. (**E**) Cell cycle. (**F**) G1 phase cell. Green fluorescence: living cells, red fluorescence: dead cells; the scale was 100 nm. The data shown are means ± S.E.M. (*n* = 3). Asterisks indicate significant differences with a two-tailed *t*-test: *** *p* < 0.001; n.s. *p* > 0.05.

We knocked down the *Cdk2* gene in BmN cells to investigate its function using CRISPR/Cas9-mediated genome editing. The mRNA expression of *Cdk2* gene was significantly down-regulated after transfection with Cas9 and sgRNA; in particular, the double-target knockdown effect was significant (Figure 6A). Down-regulating *Cdk2* did not affect cell viability (Figure 6B) but significantly affected cell proliferation (Figure 6C,D). Flow cytometry showed that the down-regulation of *Cdk2* led to a blockage of the G1 to S transition (Figure 6E). This resulted in a significant increase in the G1 phase cell ratio (Figure 6F).

## 3. Discussion

Cdk2 is a highly conserved cyclin-dependent kinase in the serine protease family that plays a key role in the key test points of the cell cycle [30,31,32,33,34,35]. Cdk2 is critical in the completion of G1 phase and the transition from G1 to S phase [36,37]. Our CRISPR/Cas9 experiments in BmN cells revealed that Cdk2 depletion specifically impairs the G1/S transition, leading to proliferative defects. These findings align with the observations in *M. musculus*, where Cdk2 deficiency disrupts prophase I progression during meiosis, resulting in sterility [38]. Similarly, in *X. laevis*, Cdk2 activity maintains meiotic arrest, with its inhibition triggering premature cell cycle resumption [27]. These cross-species parallels highlight the evolutionary conservation of Cdk2’s cell cycle regulatory functions.

Cytological studies of parthenogenesis induced by warm bath treatment in silkworm have shown that the spindle of the first meiosis division in unfertilized eggs is destroyed. Equitably separated and formed an unreduced prokaryote that can develop into a new individual with the same genotype (ZW) as the mother [39]. Typically, Cdk2 coordinates with cyclins to precisely control these cell cycle events [40]. After the unfertilized eggs of parthenogenetic lines and corresponding amphigonic lines were warmed, the parthenogenetic line had a more effective response to buffer heat stress, and cell cycle-related proteins such as histone and spindle-associated proteins were significantly reduced [41]. Cdk2 and cyclin E formed a complex that co-phosphorylates substrate factors to propel the cell through the G1/S checkpoint and successfully into the S phase [42]. The Cdk2–cyclin A complex was essential for the smooth progression of the G1 to S phase, initiating DNA replication in cells [43]. In silkworm, impaired Cdk2 activity disrupts these carefully coordinated processes during parthenogenetic induction, ultimately compromising embryonic development.

The warm bath-induced parthenogenetic process in silkworm represents a complete cloning mechanism, where oocyte division occurs without allelic recombination, resulting in offspring that maintain identical genotypes with the maternal parent [16,39]. This unique characteristic enables the maximum preservation of desirable traits across successive generations and a significant enhancement of breeding efficiency. As a model organism in lepidoptera, silkworm parthenogenesis offers an additional research value: it facilitates the conservation of superior genetic traits; improves the selection and amplification efficiency of target genes; and provides an optimal system for insect resource utilization and development. Our study elucidated the critical regulatory role of Cdk2 in warm bath-induced silkworm parthenogenesis. These findings not only provide fundamental insights into the molecular mechanisms governing parthenogenesis but also identify potential molecular targets for optimizing artificial parthenogenesis techniques and improving silkworm breeding efficiency.

In summary, our study indicated the key role of Cdk2 in the induction of warm bath for parthenogenesis in silkworm. The decrease in Cdk2 activity led to the obstruction of G1/S conversion, which affected the induction rate of parthenogenesis. This study represents a significant advancement from early phenomenological observations of artificial parthenogenesis to a molecular-level understanding of its regulation. Given Cdk2’s evolutionary conservation, these insights not only elucidate the mechanisms governing silkworm reproduction but also provide valuable implications for reproductive regulation across species. Future research should explore the specific cyclin partners and downstream effectors of Cdk2 in this context, potentially opening up new avenues for biotechnological applications in sericulture and beyond.

## 4. Materials and Methods

### 4.1. Silkworm and Cell Lines

The PLs (Wu9, Wu14) and ALs (Fengyi, 54A) were bred and provided by the Zhejiang Academy of Agricultural Sciences. Wu9 and Wu14 were bred from Fengyi and 54A by warm bath (46 °C, 18min) induction, respectively [41]. They have been bred for 30 generations and have stable characters. Silkworm eggs were incubated at 25 °C 80% RH incubator for 10 days. The instar larvae were reared on fresh mulberry leaves at 25 °C 70% RH. To break diapause, eggs were treated with 15% hydrochloric acid at 46 °C for 5 min before incubation. The BmN cell line, which originated from a *B. mori* ovary, was used [44]. Cells were maintained in TC-100 medium (LVN1013, Livning, Beijing, China) containing 10% fetal bovine serum (FBS) in 25 cm^2^ Petri dishes at 27 °C.

### 4.2. Artificially Induced Parthenogenesis

Twelve hours after emergence, the abdomen of female moths was dissected and we collected unfertilized eggs for induction [12]. The unfertilized eggs were soaked in a warm bath at 46 °C for 18 min and rapidly cooled in a room-temperature water bath at 25 °C for 3 min. The eggs were dried and placed in an incubator at 16 °C, 80% RH for 3 days. The eggs at this time corresponded to the developmental stage of sexual reproduction just after laying eggs. The eggs were immersed in 15% hydrochloric acid at 46 °C for 5 min to remove diapause and then placed in a 25 °C 80% RH incubator until hatching. After the activation of parthenogenesis, the embryo develops so that the egg color changes from yellow to dark due to serous pigmentation on the third day [45]. However, not all pigmentated eggs hatched successfully. The pigmentation and hatching rates of the eggs in each moth were calculated (*n* = 45). Pigmentation rate (%) = (number of eggs pigmentated/total number of eggs) × 100%. Hatching rate (%) = (number of eggs hatched/total number of eggs) × 100%.

### 4.3. Protein Structure and Phylogenetics Analysis

Protein structure was predicted using the online software ProtScale (https://web.expasy.org/protscale/, accessed on 20 January 2025) and SWISS-MODEL (https://swissmodel.expasy.org/, accessed on 20 January 2025) [46]. The evolutionary relation of the Cdk2 protein was inferred using the neighbor-joining method. The tests were performed by Bootstrap (1000 replicates). Evolutionary distances were computed using the Poisson correction method. Evolutionary analyses were conducted in MEGA 11 [47].

### 4.4. RNA Isolation, cDNA Synthesis, and qPCR Analysis

Total RNAs were isolated using Trizol reagent (Invitrogen, Carlsbad, CA, USA). For complementary DNA (cDNA) synthesis, 1 μg of total RNA was used with the RevertAid™ First Strand cDNA Synthesis Kit (Thermo Fisher Scientific, Waltham, MA, USA). Quantitative real-time PCR (qRT-PCR) analyses were performed using a SYBR Green Realtime PCR Master Mix (Thermo Fisher Scientific, Waltham, MA, USA). The PCR conditions were as follows: initial incubation at 95 °C for 5 min, 35 cycles at 95 °C for 15 s, and 60 °C for 1 min. The *ribosomal protein 49* (*Bmrp49*) was used as an internal control [48]. A relative quantitative method (^ΔΔ^Ct) was used to evaluate quantitative variation. The gene-specific primers used for qRT-PCR were listed in Table 1.

### 4.5. Enzyme Inhibition and Activity Detection

Healthy individuals with consistent pupation time were selected and placed at 25 °C in an 80% RH incubator. AUZ454 has been reported to be a potent and specific inhibitor of Cdk2 [29]. AUZ454 was injected into the pupa using a microsyringe. The needle is injected vertically into the middle of the third somite on the abdomen. The pupal stage of PLs (Wu9, Wu14) and ALs (Fengyi, 54A) was 13 days, and the injection was given on the 12th day (one day before emergence). DMSO: corn oil (1:9, *v*/*v*) was used as a solvent to configure different concentrations of inhibitors. According to the characteristics of inhibitors and the preliminary experimental concentration test, five appropriate concentrations were finally set for the inhibitor (20 µM, 80 µM, 160 µM, 320 µM, 1000 µM). Eggs were collected 12 h after female emergence, and enzyme activity was detected using an ELISA kit (YJ851570, YuanjuBio, Shanghai, China).

### 4.6. Ovariole and Embryo Observation

Female moths 12 h after emergence were dissected, and 8 ovarioles were separated with tweezers in insect phosphate-buffered saline (PBS). Images were captured using a camera (Canon, EOS M3, Tokyo, Japan). KOH solution (100 mL, 20%) was added to a beaker and heated to boiling. Eggs were soaked in KOH solution for 3 s and immediately transferred to 60 °C warm water for 3 s. Then, the eggs were placed in a Petri dish with 25 °C water and blown repeatedly with a plastic glue head dropper until the complete embryos were obtained. Images were captured using a microscope (TL3000 Ergo, Leica, Wetzlar, Germany).

### 4.7. mRNA Synthesis and Cell Transfection

Two single-guide RNA (sgRNA) target sites were designed according to the 5′-GGNGG-3′ principle, including ‘GGTCTCTGTGGAGAACACGTTGG’ and ‘GGTCTATTTCACTGTCACCGGGG’. sgRNA templates were synthesized based on oligonucleotides that encode the T7 polymerase binding site and were subsequently annealed to common oligonucleotides that encode the remainder of the sgRNA sequence. The reaction conditions were as previously described [49]. sgRNAs were synthesized in vitro using the MEGAscript Kit (Ambion, Austin, TX, USA). Cas9 mRNAs were synthesized using the Mmessage mMACHINE kit (Ambion, Austin, TX, USA). The sgRNA-specific primers used for plasmid construction are listed in Table 1.

sgRNAs and Cas9 mRNAs were transfected separately into BmN cells using the lipofectamine reagent (Invitrogen, Carlsbad, CA, USA) following the manufacturer’s protocol. For transfection, 500 μM of each RNA was transfected into BmN cells in a 6-well cell culture plate in triplicate. The transformed BmN cells were cultured with TC100 insect medium (LVN1013, Livning, Beijing, China) at 27 °C.

### 4.8. Cell Viability and Cycle Assay

After transfection for 48h, 100 µL of cell suspension was added to a 96-well cell culture plate containing 10 µL of CCK-8 solution (40203ES60, YEASEN, Shanghai, China) in triplicate. Then, culturing was carried out at 27.5 °C for 2 h and the absorption value at 450 nm wavelength was determined to reflect the cell viability and proliferation. Meanwhile, the living/dead cell double staining kit (CA1630, Solarbio, Beijing, China) was used to observe the ratio of living cells (green) to dead cells (red) by fluorescence staining. The cell cycle and apoptosis analysis kit (C1052, Beyotime, Shanghai, China) was used to detect cell cycle. The cells were collected by centrifuge at 1000× *g* for 3 min. In total, 1 mL of 70% ethanol was added, and then they were fixed at 4 °C for 24 h. The cells were precipitated by centrifugation at 1000× *g* for 5 min and washed twice with PBS. A total of 500 μL of propidium staining solution was added, and then they were incubated at 37 °C for 30 min without light. Red fluorescence was detected using flow cytometry (BD FACSAria™ III, Piscataway, NJ, USA) at 488 nm. The cell cycle distribution was calculated using FlowJo 7.6.5 software.

### 4.9. Statistical Analysis

GraphPad Prism 8.3.0 was used for Student’s two-tailed *t*-test analysis. Three independent replicates were used for each treatment. Means were determined, and error bars show the means ± SEM (standard error of the mean).

## Figures and Tables

**Table 1:** Specific primers.

Primer Name	Primer Sequence (5′-3′)
qRT-PCR	
BmCdk2-F	TACCGTGCACCAGAAATCCT
BmCdk2-R	GCTCGGAAGTCAGGAAGTCT
BmRP49-F	TCAATCGGATCGCTATGACA
BmRP49-R	ATGACGGGTCTTCTTGTTGG
Plasmid construction	
sgRNA-F1	TAATACGACTCACTATAGGTCTCTGTGGAGAACACGTGTTTTAGAGCTAGAAATAGCAA
sgRNA-F2	TAATACGACTCACTATAGGTCTATTTCACTGTCACCGGTTTTAGAGCTAGAAATAGCAA
sgRNA-R	AGCACCGACTCGGTGCCACTTTTTCAAGTTGATAACGGACTAGCCTTATTTTAACTTGCTATTTCTAGCT

## Data Availability

The data that support the findings of this study are available on request from the corresponding author.
